# Effects of parents and Brown-headed Cowbirds (*Molothrus ater*) on nest predation risk for a songbird

**DOI:** 10.1002/ece3.411

**Published:** 2012-11-08

**Authors:** Quresh S Latif, Sacha K Heath, John T Rotenberry

**Affiliations:** 1 Department of Biology, University of CaliforniaRiverside, California; 2 PRBO Conservation SciencePetaluma, California; 3 College of Biological Sciences, University of MinnesotaSaint Paul, Minnesota

**Keywords:** Mono Lake, nest parasitism, nest predation, nest survival, parental behavior, population ecology, predator–prey interactions, *Setophaga petechia*, Yellow Warbler

## Abstract

Nest predation limits avian fitness, so ornithologists study nest predation, but they often only document patterns of predation rates without substantively investigating underlying mechanisms. Parental behavior and predator ecology are two fundamental drivers of predation rates and patterns, but the role of parents is less certain, particularly for songbirds. Previous work reproduced microhabitat-predation patterns experienced by Yellow Warblers (*Setophaga petechia*) in the Mono Lake basin at experimental nests without parents, suggesting that these patterns were driven by predator ecology rather than predator interactions with parents. In this study, we further explored effects of post-initiation parental behavior (nest defense and attendance) on predation risk by comparing natural versus experimental patterns related to territory density, seasonal timing of nest initiation, and nest age. Rates of parasitism by Brown-headed Cowbirds (*Molothrus ater*) were high in this system (49% nests parasitized), so we also examined parasitism-predation relationships. Natural nest predation rates (NPR) correlated negatively with breeding territory density and nonlinearly (U-shaped relationship) with nest-initiation timing, but experimental nests recorded no such patterns. After adjusting natural-nest data to control for these differences from experimental nests other than the presence of parents (e.g., defining nest failure similarly and excluding nestling-period data), we obtained similar results. Thus, parents were necessary to produce observed patterns. Lower natural NPR compared with experimental NPR suggested that parents reduced predation rates via nest defense, so this parental behavior or its consequences were likely correlated with density or seasonal timing. In contrast, daily predation rates decreased with nest age for both nest types, indicating this pattern did not involve parents. Parasitized nests suffered higher rates of partial predation but lower rates of complete predation, suggesting direct predation by cowbirds. Explicit behavioral research on parents, predators (including cowbirds), and their interactions would further illuminate mechanisms underlying the density, seasonal, and nest age patterns we observed.

## Introduction

Predation is the main cause of nest failure for many bird species (Martin 1993), and nest survival is an important component of fitness (Lack 1966; Saether and Bakke 2000). Consequently, predation of nests has shaped the evolution of avian behaviors such as nest-site selection and parental attendance (Ghalambor and Martin 2002; Peluc et al. 2008), life history characteristics such as clutch size (Martin 1995), and morphological traits such as egg color (Kilner 2006). Nest predation also shapes population growth (Saether and Bakke 2000) and community structure by favoring nest-site diversification to reduce competition for predator-free space (Lima and Valone 1991). Therefore, ornithologists study nest predation to better understand the evolution and ecology of birds.

An understanding of how and why nest predation occurs requires examination of the predation process (Lahti 2009). Nest predation involves interaction between predator and prey, so ecological traits of predators, namely their abundance and behavior, determine predation risk (Thompson 2007). Accordingly, several studies link predator ecology with predation rates and patterns (Schmidt and Ostfeld 2003a,b; Sperry et al. 2008; Weatherhead et al. 2010). Nesting parent birds also influence predation risk by deciding where to nest (Martin 1998; Davis 2005; Peluc et al. 2008; Latif et al. 2012), modulating activity at the nest and consequently the cues used by predators (Ghalambor and Martin 2002), and defending their nests when predators attack (Blancher and Robertson 1982; Hogstad 2004). For small songbirds, the importance of nest-site selection is well recognized (reviewed by Lima 2009), which can influence predation patterns observed at natural nests (Schmidt and Whelan 1999a; Latif et al. 2012).

The extent to which small songbirds can influence predation risk following nest initiation is less certain. Parental and nestling activity (e.g., begging) at the nest can attract predators and increase predation risk (Martin et al. 2000), so parents modulate activity at the nest to avoid increasing risk (Ghalambor and Martin 2002; Eggers et al. 2008). Birds can further reduce predation risk by defending their nests, either actively (Blancher and Robertson 1982; Hogstad 2004) or passively (Halupka 1998). Small birds exhibit various defensive behaviors (Ghalambor and Martin 2002; Colombelli-Négrel et al. 2010; see also review by Montgomerie and Weatherhead 1988), but some have doubted the efficacy of such behavior against certain predators (e.g., nocturnal predators; Bradley and Marzluff 2003). Nevertheless, studies do provide evidence for effective nest defense even by small songbirds (initially reviewed by Martin 1992; see also Pietz and Granfors 2005), with intensity and efficacy dependent on food availability (Duncan Rastogi et al. 2006), nest-site quality (Remeš 2005), or predator type (Schmidt and Whelan 2005).

**Figure 1:**
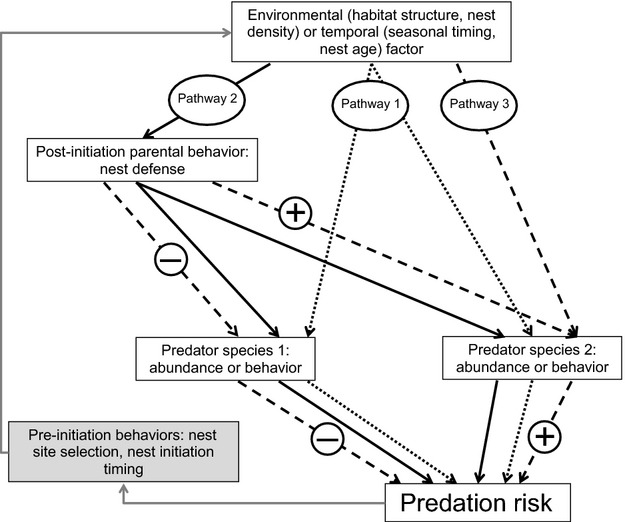
Pathways by which environmental or temporal factors could correlate with avian nest predation risk. Predator ecology could exclusively drive patterns (pathway 1). Alternatively, parental activity at the nest (i.e., post-initiation activity; nest defense or parental visitation rates) could modulate patterns. Parental behaviors affecting predation risk could vary (pathway 2), or parental interactions could vary among predator species that correlate differently with environmental or temporal factors (pathway 3; for this pathway, environmental/temporal factors affect predator 2, which parents attract, but not predator 1, which is parents deter). Pre-initiation parental behaviors (e.g., nest-site selection or nest-initiation timing) can respond to predation patterns and influence the environments or time periods in which nests are exposed to predation. Preinitiation behaviors are a step removed, however, from the fundamental mechanistic drivers of predation risk.

By definition, nest predation involves predators, but determining the extent to which parents are involved can help narrow the range of mechanisms and thus causal factors underlying a pattern of interest. Patterns could arise exclusively from variation in predator ecology, namely their abundance or behavior (Thompson 2007). Parents can adaptively respond to these patterns when selecting nest sites, in which case parents can influence observed patterns (Schmidt and Whelan 1999a; Latif et al. 2012) but leaving predators as the fundamental drivers of predation risk (pathway 1, Fig. 1). Alternatively, post-initiation parental behavior (i.e., nest defense or nest activity) can modulate predation-risk patterns if parental behavior itself varies (pathway 2, Fig. 1), or if parental interactions vary among ecologically different predator species (pathway 3, Fig. 1). If predation patterns are driven exclusively by predator ecology, information regarding alternative prey for predators (Schmidt and Ostfeld 2003a) or predator-habitat relationships (Chalfoun et al. 2002; Schmidt and Ostfeld 2003b) could illuminate underlying mechanisms. Alternatively, if parental behavior modulates observed patterns, food availability for nesting birds (Martin 1992), the presence of conspecifics (Hogstad 1995; Sperry et al. 2008), or factors influencing how parents respond to predators, and vice versa, may also be relevant.

Experimental nests (i.e., artificial nests) provide a potentially useful tool for examining the role of post-initiation parental behavior as a driver of nest predation patterns. Experimental nests have been used widely to study nest predation (reviewed by Major and Kendal 1996), but experimental predation rates and patterns often differ from those experienced by natural nests raising questions about the relevance of experimental-nest data (Faaborg 2004; Moore and Robinson 2004). Among the major reasons suspected for these differences are that experimental nests lack parents (Weidinger 2002). Analysis of the differences in experimental versus natural predation rates and patterns could therefore suggest how parents contribute to predation risk (Weidinger 2002).

**Figure 2:**
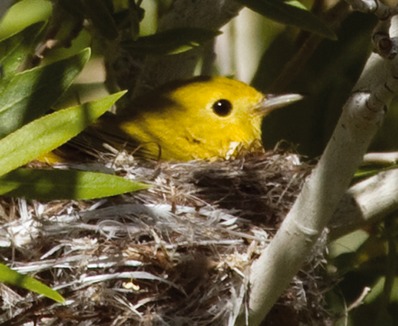
Photograph of incubating female Yellow Warbler along Rush Creek, Mono Lake Basin, CA.

We studied the mechanistic pathways underlying predation rates and patterns experienced by a population of Yellow Warblers (*Setophaga petechia*; Fig. 2) over an 8-year period (2001–2008). Previous work in this study system documented the adaptive significance of nest microhabitat selection for avoiding predation, the principal cause of nest failure. Parents adaptively favored nest-site concealment levels associated with reduced predation risk (Latif et al. 2012), but maladaptively favored microhabitat patch compositions associated with elevated predation risk (Latif et al. 2011). Experimental nests placed in microhabitats also occupied by natural nests recorded similar microhabitat-predation patterns, suggesting predator ecology as the main driver of microhabitat-related predation patterns (pathway 1, Fig. 1). Nest-survival rates were highly variable, suggesting a possible factor contributing to the persistence of maladaptive nest microhabitat preferences; non-microhabitat sources of variability might reduce the contribution of microhabitat-predation patterns (i.e., % variance explained) to overall fecundity and thus reduce the cost of maladaptive nest-site preferences. We therefore expected a closer examination of non-microhabitat correlates of predation rates to provide some context for understanding previous work by further illuminating additional factors contributing to predation risk. Studies elsewhere have identified breeding densities (Schmidt and Whelan 1999b; Hogstad 1995; Perry et al. 2008), seasonal timing, and nest age (Nur et al. 2004, Grant et al. 2005) as potentially important correlates of predation rates, so we were interested in their importance here. Additionally, Yellow Warblers in this system were heavily parasitized by the Brown-headed Cowbird (*Molothrus ater*; hereafter cowbird), which can affect nest predation in various ways (Arcese et al. 1996; Peer and Bollinger 2000; Tewksbury et al. 2002; Hoover and Robinson 2007), so we were also interested in parasitism relationships with predation risk.

We examined whether parents modulated nest predation patterns related to breeding territory density, seasonal timing, and nest age by comparing patterns observed at natural nests to those observed at experimental nests without parents. We first analyzed patterns across the entire study period to identify those generally experienced by natural nests. We then compared natural patterns to those recorded at experimental nests during 2 years when both were monitored concurrently and across a similar spatial extent. Our analysis accounted for differences between natural and experimental nests other than the presence of parents, allowing us to tease apart potential mechanistic pathways underlying observed patterns (i.e., pathway 1 vs. pathways 2 or 3; Fig. 1). Additionally, we compared overall predation rates to examine the relative influence of parental defense (expected to reduce predation rates for natural nests) versus nest activity (expected to elevate predation rates) in determining natural predation rates. Finally, we analyzed predation relationships with brood parasitism allowing consideration of how cowbirds might affect nest predation risk and patterns.

## Materials and Methods

### Study system

**Figure 3:**
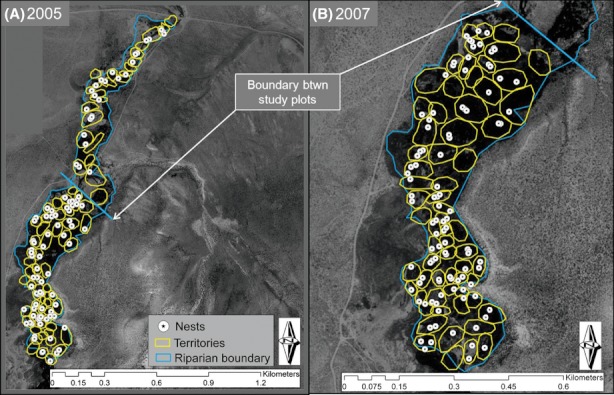
Mapped Yellow Warbler territories superimposed on an aerial photograph of Rush Creek during two example years of the study period (2005 and 2007). The distribution of territories varied among years, but areas containing the highest and lowest densities were similar across years. In 2001–2005, nests were monitored at two study plots (A), whereas in 2006–2008, only the upper (southern) study plot was monitored (B).

We studied nest predation for a population of Yellow Warblers from 2001 to 2008 along the lower reaches of Rush Creek, the largest tributary of Mono Lake, east of the Sierra Nevada in California, USA (2020 m, 38°04′N, 119°10′W). The Yellow Warbler is an open-cup, shrub, and tree-nesting neotropical migrant passerine species that breeds mainly in riparian habitats across North America (Lowther et al. 1999). Male Yellow Warblers arrive and establish territories along Rush Creek in early May. Females select nest sites from within these territories, initiating nests from late May to early July. From 2001 to 2005, we collected data from two Rush Creek study plots totaling 39 ha and two stream-kilometers as part of a multispecies demographic monitoring program (Heath et al. 2006; Fig. 3A). From 2006 to 2008, we continued studying Yellow Warblers at one of these plots (20 ha, 1 stream-kilometer, Fig. 3B), during which time we also monitored experimental nests. Three species of willow (*Salix exigua*, *S. lucida*, *S. lutea*) were the principal woody plants within this study area, but substantial stands of Woods' rose (*Rosa woodsii*) and big sagebrush (*Artemisia tridentata*) were also present (see Latif et al. 2011 for detailed habitat description).

Numerous predator species prey on open-cup nests including those of Yellow Warblers in the Mono Basin. Confirmed nest predators along Rush Creek include garter snakes (*Thamnophis* sp.), gopher snakes (*Pituophis catenifer*), mice (Muridae/Cricetidae), chipmunks (*Tamias* sp.), raccoons (*Procyon lotor*), weasels (*Mustela* sp.), Western Scrub-Jay (*Aphelocoma californica*), Black-billed Magpie (*Pica hudsonia*), wrens (Troglodytidae), and Bullock's Oriole (*Icterus bullockii*; Latif et al. In press). In addition, cowbirds parasitized 49% of Yellow Warbler nests in our study area (*n* = 683 nests; PRBO and Q. S. Latif unpubl. data) and are confirmed nest predators (Latif et al. In press).

### Field work

#### Nest searching and monitoring of natural nests

We searched for Yellow Warbler nests during the breeding season (1 May–31 July, 2001–2008). We also mapped season-long observations of territorial behavior (e.g., singing, countersinging, simultaneous nesting) to identify distinct breeding territories for unmarked Yellow Warblers. We located as many nesting attempts for as many territories as possible (Martin and Geupel 1993). We found nests for 70–94% of territories in any given year (e.g., Fig. 3), so we are confident that the nests found adequately sampled the study population.

Once located, we recorded the contents of each nest once every 3.4 ± 1.1 (SD) days until they failed or fledged young. We considered nests failed if we observed one of three scenarios: (1) no remaining Yellow Warbler eggs or nestlings in the nest prior to the earliest possible fledge date, (2) nest abandonment by the parents, or (3) eggs remaining unhatched more than 8 days past the normative incubation period (10.4 ± 1.2 [SD] days after clutch completion; *n* = 45 nests whose clutch completion and hatch timings were known to the day). We attributed nest failure to predation given scenario 1 or when predation was directly observed. We considered nests that survived to a potential fledging age (9.8 ± 0.9 days from hatching; *n* = 29 nests whose hatch and fledge timings were known to the day; Q. Latif and PRBO Conservation Science unpubl. data derived from Mono Lake birds) successful or depredated based on additional field observations. For example, direct observation of fledglings or parents carrying food shortly after nest termination indicated success, whereas initiation of new attempts coupled with no apparent food carries indicated failure (Weidinger 2007). We used standard precautions to avoid attracting predators to nests (Martin and Geupel 1993). During each visit, we determined the age of nestlings by comparing them to photographs of nestlings of known age. Additionally, in 2008, we candled eggs in the field (Lokemoen and Koford 1996) and determined egg age using comparisons with images from candling known-age eggs. We measured microhabitat structure at each nest site once nests became inactive using protocols described in detail elsewhere (Latif et al. 2011, 2012).

#### Experimental-nest placement and monitoring

**Figure 4:**
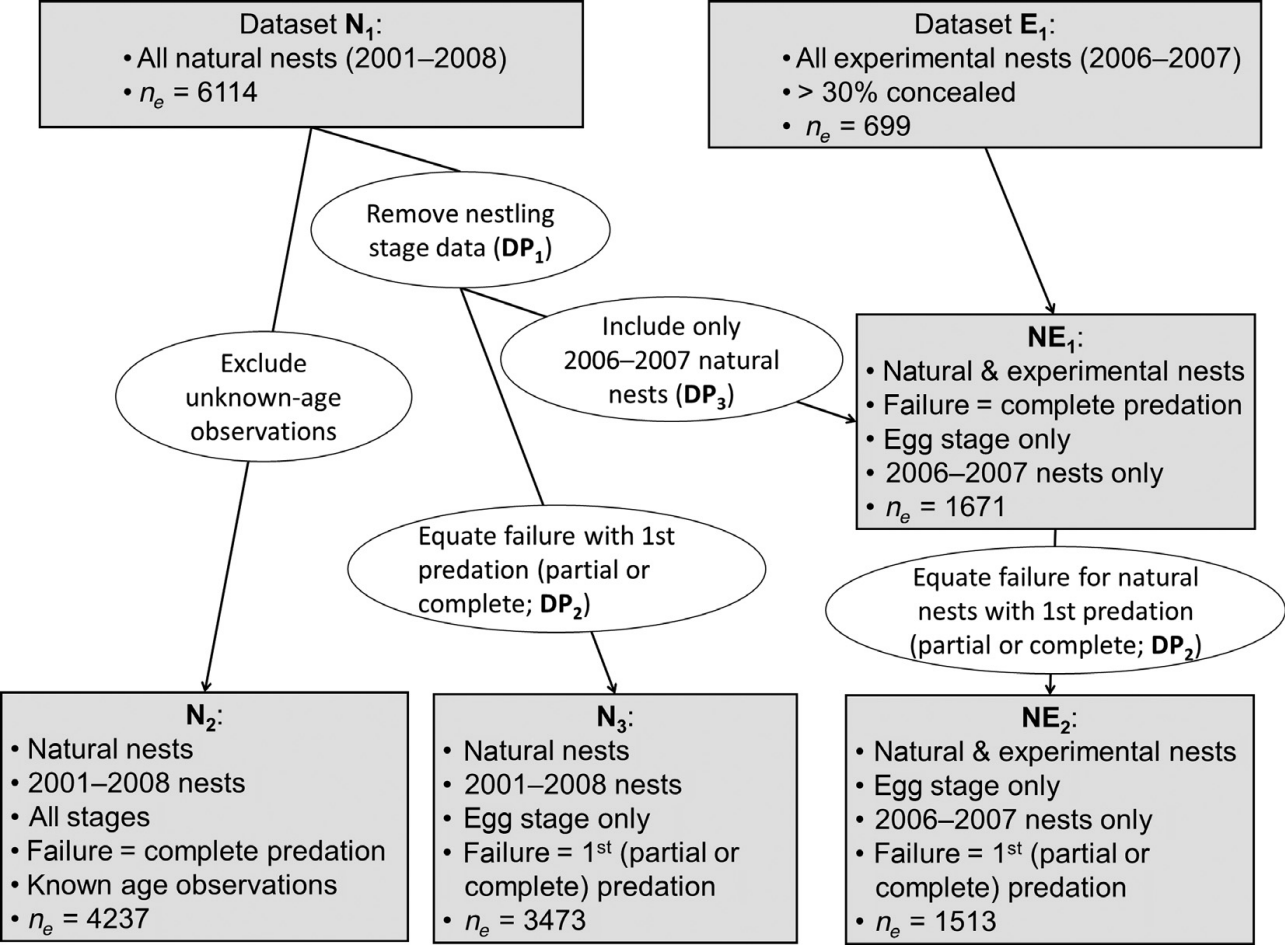
Flowchart showing the data processing steps (DP_1–4_) used to compile the datasets (N_1–3_, NE_1–2_, and E_1_) and the resulting structure of datasets analyzed in this study. *n_e_* = number of observation days.

Experimental nests consisted of previously used Yellow Warbler nests each containing one passerine egg (obtained from captive Zebra Finches [*Taeniopygia guttata*] and stored following established protocol to avoid spoilage until deployed in the field; DeGraaf and Maier 2001) and one clay egg. We shaped clay eggs from modeling clay, approximating the size and shape of real eggs (see photo in Latif et al. 2012). Clay eggs recorded predator-specific bite impressions analyzed elsewhere (Latif et al. 2011, 2012). We placed experimental nests in shrubs typically occupied by natural nests (willow or rose) and monitored them concurrently with natural nests (25 May–22 July) in 2006–2007 and within the same spatial extent as natural nests during those years (Fig. 3B). To accommodate a separate study (Latif et al. 2012), we monitored experimental nests across extended concealment and height ranges beyond what natural nests typically occupied, although we did place 49% of experimental nests within the natural range (>75 cm, and from 30% to 80% concealed). We excluded data from 29 experimental nests in sites <30% concealed from all analyses in this study, as these sites were atypical for natural nests and associated with atypical predation rates (Latif et al. 2012). Thus, all remaining experimental nests were either within the natural microhabitat range (62% of nests) or experienced predation rates similar to those recorded within the natural range (38% of nests). We monitored experimental nests using the protocol for monitoring natural nests until depredation (i.e., eggs were damaged or disappeared) or for 13 days (i.e., the Yellow Warbler laying and incubation periods; PRBO unpubl. data). We compiled nest-monitoring data into various datasets (Fig. 4) for specific analyses described below.

### Data analysis

#### Nest-survival models

We analyzed nest-survival rates using logistic exposure, a generalized linear model that employs a logit link function with a binomial distribution to model daily survival rate (DSR) as a function of explanatory variables (Shaffer 2004). The sampling unit was the *observation interval* (the period between nest checks), models accounted for interval length allowing analysis of DSR, and nest outcomes (success vs. failure) during each interval were assumed independent. Logistic exposure models (hereafter DSR models) were fitted with PROC GENMOD (SAS 9.1; SAS Institute, Cary, NC, USA) to data from nests observed active with at least one Yellow Warbler egg or live nestling. We excluded observation intervals during which nests failed for reasons other than predation (e.g., scenarios 2 or 3 above; 25% of failures), making predation rates equal to one minus survival rates. We considered nests failed either when completely depredated (i.e., when no host eggs or young remained) or when first depredated (either partially or completely, where partial predation = some but not all host eggs or young being depredated) depending upon the analysis (described further below). We defined failure or success based solely on the fate of host contents. Thus, we considered nests completely depredated when all host contents were lost even if viable cowbird eggs or nestlings remained.

All DSR models described nest survival as a function of one or more explanatory variables: breeding territory density, within-season nest-initiation timing, nest age, and parasitism status (whether the nest was parasitized by a cowbird). We generated nest-specific territory density values by counting the number of digitized territories whose boundaries intersected a 150-m radius buffer centered on each nest using GIS software (ArcGIS 9.2, ESRI 2006), and then dividing the number of territories by the area (ha) of the buffer (Density = no. territories/ha) excluding any area outside the riparian corridor or outside the study plot. Riparian edges were easily identifiable from aerial photographs. Distance to habitat edge could correlate with nest predation (Andren and Angelstam 1988; Paton 1994; but see Tewksbury et al. 1998) and could be confounded with nest density if nests are sparser along edge versus core habitat. A strong relationship between territory density and distance to edge was not apparent from visual inspection of territory maps (Fig. 3). We initially calculated densities with 50-, 100-, and 150-m radius buffers, but density values were correlated (*r* ≥ 0.62, *n* = 860) among buffer sizes and the 150-m-based values covaried the strongest with nest-survival rates, so we used 150-m-buffer values. We described nest-initiation timing as clutch completion date (day-of-year). We described nest age continuously (Age = days from clutch completion; clutch completion age = 0; laying ages were negative), as a two-class variable (Stage = egg or nestling), or as a three-class variable (Stage = laying, incubation, or nestling). We scored nest parasitism status at each observation interval; a nest containing at least one cowbird egg or nestling was parasitized. Parasitism correlations with predation rates suggested predation by cowbirds (see Results and Arcese et al. 1996; Hoover and Robinson 2007), so interactions between parasitism and predation patterns suggested whether cowbirds might be driving these patterns. We considered nonlinear patterns using quadratic (e.g., Date^2^ = Date + Date^2^) or cubic parameters (e.g., Age^3^ = Age + Age^2^ + Age^3^).

We considered the potential for confounding effects by including additional explanatory variables in DSR models or examining correlations between variables of interest with potentially confounding variables. Depending upon the analysis, explanatory variables described above sometimes controlled for confounding effects (e.g., date and density effects for age-related analysis and vice versa). Additionally, all DSR models included Year (a categorical variable) and a microhabitat variable, PC1 (calculated for a separate study; Latif et al. 2011), to control for confounding effects not of direct interest in this study. PC1 was the first component generated from a principal components analysis applied to measurements of 5-m radii patches centered on the nest site describing overhead cover (based on densitometer measurements), percent coverages of three shrub types (willow [*Salix* spp.], rose [*R. woodsii*], and nonriparian shrubs [mainly *A*. *tridentata*]) and willow stem counts. PC1 correlated positively with willow variables, and so described a willow–nonwillow microhabitat gradient that also correlated positively with nest predation rates (NPR; Latif et al. 2011). We also considered confounding effects of concealment (percent of the nest-cup hidden by surrounding vegetation; measured via ocular estimation) and Height (the distance [cm] from the ground to the bottom of the nest-cup) (for further details on measurement protocols and observer training used to standardize height and concealment, see Latif et al. 2012) mainly by examining intercorrelations with variables of interest. Additionally, models applied to experimental-nest data (described below) explicitly included height.

We used information theory (Burnham and Anderson 2002) to examine the statistical support for effects of interest via model comparison. We calculated model weights (*w*_*i*_) from AIC_c_-differences (AIC_c_ = Akaike Information Criterion corrected for small-sample bias) between a given model and the best-fit model (lowest AIC_c_) in a given model set. Evidence ratios(ER = Σ*w*_models-with-effect_/Σ*w*_models-without-effect_) quantified the relative support for effects of interest. We calculated NPR using top DSR models (NPR = 1 − DSR^23^ for the entire natural-nest period or 1 − DSR^13^ for the egg period, where exponents are nest-period lengths in days) assuming mean values for nontarget variables calculated for the data to which models were fitted. We applied the delta method to logit estimates to calculate standard errors and 95% confidence intervals for nest survival (Powell 2007). We tested the goodness-of-fit of models using *ĉ* (χ^2^_GOF_/degrees-of-freedom) for maximally parameterized models, where *ĉ* > 1 indicated some lack-of-fit and *ĉ* > 4 indicated unacceptably poor model fit (Burnham and Anderson 2002). Given evidence of lack-of-fit (*ĉ* > 1), we also compared model-based estimates (predicted values) to class-based estimates (analogous to observed data for linear regression) of predation rates to further examine model fit (Shaffer and Thompson 2007). Additionally, we inflated DSR variances by *ĉ* when *ĉ* > 1. With finite sample sizes, deviance (upon which *ĉ* is based) overestimates dispersion in residuals (Dinsmore et al. 2002), but more accurate estimates of overdispersion are unavailable for nest-survival data, so our estimates of variance should be considered conservative.

#### Analyses of natural nest predation patterns

We analyzed natural nest predation patterns using data from all years of the study (2001–2008; Datasets N_1–2_, Fig. 4) to identify general patterns characteristic of the study system across a larger spatial and temporal extent. We analyzed density- and date-related patterns using all available data from natural nests (N_1_, Fig. 4) to which we fitted and compared models representing all possible combinations of Date, Date^2^, Density, Density^2^, and Parasitism effects. All these models contained Year, PC1, and Stage_Egg-or-Nestling_ to control for confounding effects. We analyzed age-related patterns using a dataset that only included observations of nests during which age was known in the field, which excluded incubation-period observations from nests found after laying unless eggs were candled (N_2_, Fig. 4). To these data, we fitted and compared five models containing one of five candidate age effects (Age, Age + Age^2^, Age + Age^2^ + Age^3^, Stage_Egg-or-Nestling_, or Stage_Laying-Incubation-or-Nestling_) along with PC1, Year, and well-supported parameters identified from the previous analysis (Date^2^, Density, and Parasitism) to control for confounding effects.

#### Comparison of natural versus experimental NPR and patterns

We compared natural versus experimental predation rates to (1) examine whether parents drive observed nest predation patterns (i.e., distinguish pathways 2 or 3 from pathway 1 in Fig. 1), and (2) examine the relative importance of nest defense versus nest activities that attract predators in determining overall predation risk. We used a series of data processing steps (DP_1–3_, Fig. 4) to compile datasets that included natural- and experimental-nest data and controlled for differences between nest types other than the presence of parents (N_3_, NE_1–2_, and E_1_; Fig. 4). Experimental nests differed from natural nests by (1) never containing nestlings, (2) they could never be partially depredated, (3) they were only monitored during 2 years, (4) they occupied a wider microhabitat range than natural nests, and (5) they were never parasitized by cowbirds. We relied principally on models fitted to a dataset (NE_2_) that controlled for most of these differences. This dataset only included 2006–2007 natural-nest data (DP_2_), excluded nestling-period data (DP_1_), and excluded experimental nests <30% concealed (justified above). Additionally, we coded natural nests that were partially depredated (i.e., some but not all host eggs were depredated) as failed upon the first incidence of partial predation (DP_3_). All DSR models fitted to these data included a Nest-Type parameter (experimental vs. natural) and spatiotemporal × Nest-Type interaction parameters. ERs for interaction parameters quantified support for differences between experimental versus natural predation patterns. Data exclusion limited our statistical power to obtain support for spatiotemporal × Nest-Type interactions. We therefore also analyzed more inclusive datasets that controlled for fewer differences between nest types but afforded more statistical power (N_3_ and NE_1_). We considered whether differences in parasitism status (difference 5) could have caused differences between natural and experimental predation rates and patterns by comparing Parasitism and Parasitism-spatiotemporal interaction effects across relevant datasets (N_1_, N_3_, and NE_1_; described further below). We fitted models that explicitly included height as an explanatory variable (i.e., controlled for nest-height-related confounding effects) to experimental-nest data only (E_1_) for qualitative comparisons of predation patterns to supplement formal comparisons. Finally, when analyses described above (i.e., comparison of models with multiple continuous explanatory variables) failed to support effects of interest but we suspected low statistical power due to scarce data (i.e., age-effect analysis results), we examined estimates from class-based models (i.e., analogous to scatter plots of continuous data; Shaffer and Thompson 2007) to see if the data suggested any trends that might be better supported with larger sample sizes.

#### Predation and cowbird parasitism

We compared predation rates for parasitized versus nonparasitized nests to identify cowbird effects on predation. Cowbirds can affect predation in various ways for various reasons. Especially loud-begging cowbird nestlings can attract predators themselves (Dearborn 1999; Hoover and Reetz 2006) or elicit greater parental activity (Dearborn et al. 1998; Hannon et al. 2009), or parasitism can elicit parental-defense behaviors that attract predators (Tewksbury et al. 2002). Cowbirds also depredate nests directly in conjunction with their parasitic activities. They may depredate nonparasitized nests either to create new parasitic opportunities or to “retaliate” in response to host rejection of parasitic eggs (Arcese et al. 1996; Hoover and Robinson 2007). They also partially depredate parasitized clutches to enhance incubation efficiency (Peer and Bollinger 2000) or procure optimal provisioning rates for their nestlings (Kilner et al. 2004). We expected indirect effects of parasitism on predation risk to cause elevated predation rates at parasitized nests. In contrast, direct predation by cowbirds should result in more complete predation of nonparasitized nests coupled with more partial predation of parasitized nests. We compared parasitism–predation relationships across various datasets (N_1_, N_3_, and NE_1–2_, Fig. 1) to determine the relative importance of direct predation by cowbirds versus indirect effects on overall predation risk.

## Results

### Predation patterns at natural nests

From 2001 to 2008, we observed 683 Yellow Warbler nests with at least one egg or nestling. Of these, 459 nests (67.2%) failed, of which 395 (86.1% of failed nests, 57.8% of total) were depredated.

**Figure 5:**
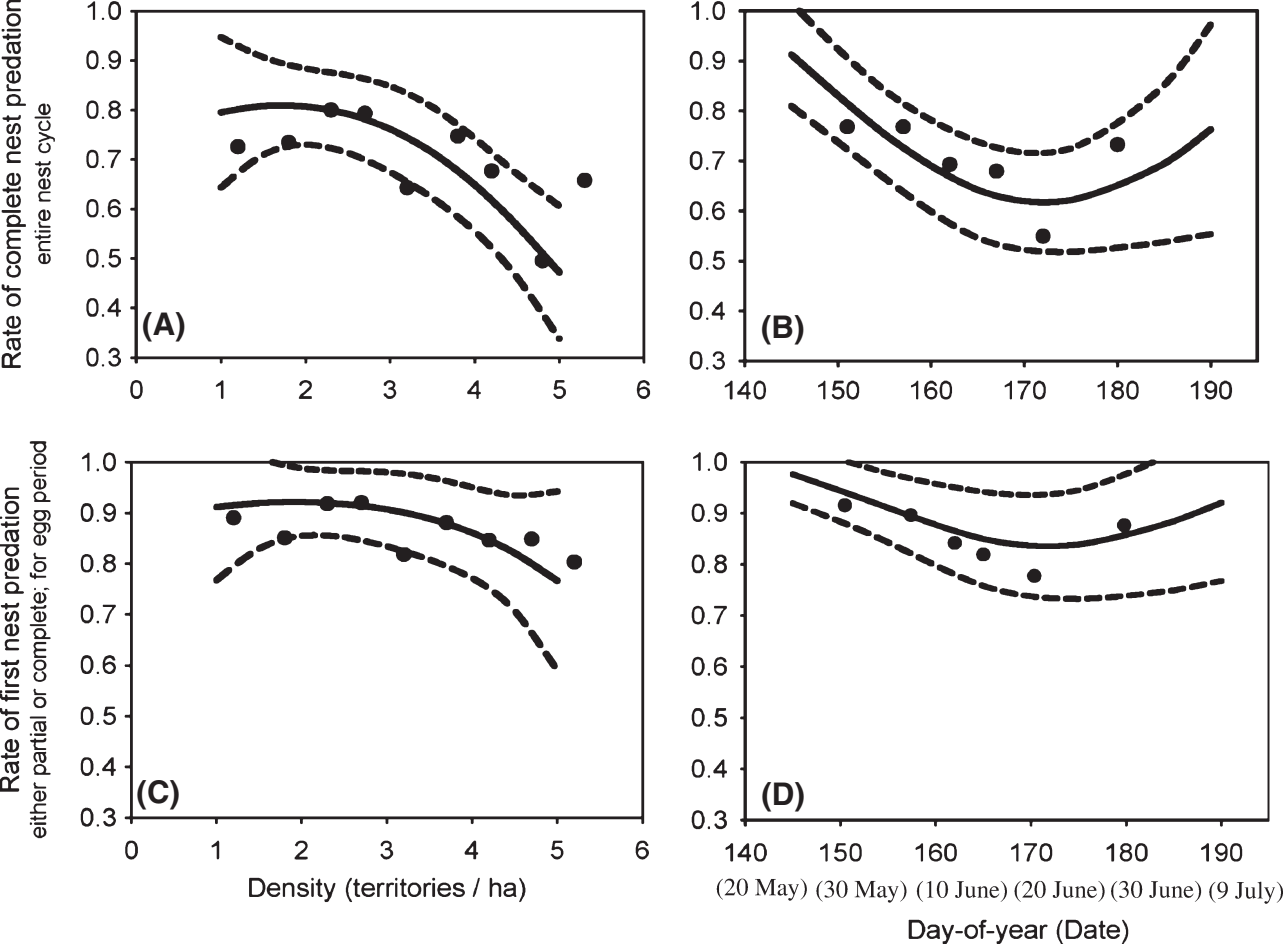
Spatiotemporal patterns in natural nest predation rates (NPR; 2001–2008). Model-based NPR estimates for the entire nest period along breeding territory density (A) and Date (B) axes were calculated using M1, Set 1, Table 1 and for the egg period when equating nest failure with first partial or complete predation (C, D) using M1, Set 2, Table 1 (parameter estimates in Table 2). Model-based estimates assume mean values for nontarget explanatory variables (Year = 0.125 for each level, PC1 = 0.2, Stage = 0.4, Parasitism = 0.5, Date = 164 for A and C, Density = 3 for B and D). Class-based NSR estimates (dots) along continuous axes are plotted at mean values for observations within each class. Class-based estimates are for assessing model fit (i.e., akin to plots of raw data alongside estimates from linear regression models; Shaffer and Thompson 2007). Dotted lines show 95% confidence bands. Variances for predation rates were calculated using the delta method (*var*(NPR) = *var*(NSR) = period^2^ × (DSR^2(period−1)^) × *var*(DSR); Powell 2007) and inflated by *ĉ* (1.8 for A and C; 2.9 for B and D). DSR, daily survival rate.

Nests were least likely to be depredated when initiated mid-seasonally (approximately 13 June) and in areas of greatest territory density. Of models fitted to natural-nest data from all years, the model with all possible effects was best supported (Model 1 [M1], Set 1, Table 1; ERs for Density^2^ and Date^2^ effects > 100). Territory densities surrounding nests varied from 0.6 to 5.8 (mean = 3.1 ± 1.2 [SD]; *n* = 683) territories/ha. Nests in the least-populated areas were approximately 1.6 times as likely to be depredated as nests in the most densely populated areas (Fig. 5A). The mean clutch completion date was day 164 ± 10 (approximately 13 June) and the modal clutch completion date was 156 (approximately 5 June). Nests whose clutches were completed in late May or early June were 1.3–1.5 times as likely to be depredated as nests initiated in mid-June (Fig. 5B; for model parameter estimates, see Table 2). Predation rates declined with nest age, and nonparasitized natural nests were completely depredated more frequently but partially depredated less frequently than parasitized nests (see details below).

**Table 1:** Models describing natural nest survival patterns for Yellow Warblers along Rush Creek (2001–2008)

Model set no., Dataset used	Model no.	Model	*K*	−LL	Δ_*i*_	*w*_*i*_
Model set 1, Dataset N_1_	1	Parasitism + Date^2^ + Density^2^	15	846.8	0.0	0.65
	2	Parasitism + Date^2^ + Density	14	849.2	2.9	0.16
	3	Date^2^ + Density^2^	14	849.4	3.3	0.12
	4	Date^2^ + Density	13	851.8	6.1	0.03
		***				
	18	Null model (Year + PC1 + Stage_Egg-or-Nestling_)	10	869.43	35.3	<0.01
	19	Constant survival	1	907.03	92.5	<0.01
Model set 2, Dataset N_3_	1	Date^2^ + Density^2^	13	683.2	0.0	0.29
	2	Date^2^ + Density	12	684.2	0.0	0.29
	3	Parasitism + Date^2^ + Density^2^	14	683.1	1.9	0.11
	4	Parasitism + Date^2^ + Density	13	684.2	2.0	0.11
	5	Date + Density^2^	12	686.2	4.0	0.04
	6	Date + Density	11	687.2	4.1	0.04
	7	Density	10	688.6	4.8	0.03
	8	Density^2^	11	687.6	4.9	0.03
	9	Parasitism + Date + Density^2^	13	686.1	5.8	0.02
		***				
	15	Null model (Year + PC1)	9	692.6	10.8	<0.01
		***				
	19	Constant survival	1	708.0	25.6	<0.01

*K* = number of model parameters, −LL = −Log-likelihood, Δ_*i*_ = ΔAIC_c_, *w*_*i*_ = AIC_c_ weights. Model sets in this table included all possible combinations of Date, Density, and Parasitism effects (19 models; all models included parameters to control for confounding effects: Year and PC1 for both model sets, and Stage_Egg-or-Nestling_ for model set 2), but only the top models for which ∑*w*_*i*_ > 0.95 are shown. *** indicates where additional models occurred but are not presented. Null models (confounding effects only) and constant survival models are also shown for comparison. Model sets were fitted to either a dataset sampling the entire nest cycle and equating nest failure with complete predation (N_1_, Fig. 4) or a dataset sampling the egg period and equating nest failure with first partial or complete predation (N_3_, Fig. 4).

Date^2^ = Date + Date^2^; Density^2^ = Density + Density^2^.

*ĉ* = 1.8 for M1, Set 1; *ĉ* = 2.9 for M3, Set 2.

**Table 2:** Parameter estimates (β ± SE) for selected models used to infer nest-survival patterns for Yellow Warblers along Rush Creek (2001–2008)

	Natural nests (Table 1)			
		
Parameters	Date, density, parasitism patterns (M1, Model set 1)^1^	Date and density patterns (M1, Model set 2)^1^	Egg-period parasitism relationship (M3, Model set 2)^1^	Model describing age-related pattern (M1, Table 5)
Density	0.25 ± 0.05^2^	0.16 ± 0.09^2^	0.16 ± 0.09	0.19 ± 0.06
Density^2^	0.09 ± 0.04^2^	0.06 ± 0.07^2^	0.06 ± 0.07	n/a
Date	0.019 ± 0.008^2^	0.015 ± 0.010^2^	0.015 ± 0.010	0.021 ± 0.009
Date^2^	−0.0013 ± 0.0006^2^	−0.0011 ± 0.0007^2^	−0.0011 ± 0.0007	−0.0011 ± 0.0007
Parasitism	0.26 ± 0.15^2^	n/a	−0.03 ± 0.20^2^	0.12 ± 0.16
Stage_Egg/Nestling_	0.83 ± 0.19	n/a	n/a	n/a
Age	n/a	n/a	n/a	0.04 ± 0.01^2^
PC1	−0.16 ± 0.08	−0.16 ± 0.11	−0.16 ± 0.11	−0.18 ± 0.09
Year = 2001	0.60 ± 0.35	0.23 ± 0.44	0.23 ± 0.44	0.57 ± 0.35
Year = 2002	0.39 ± 0.34	0.32 ± 0.48	0.32 ± 0.48	0.28 ± 0.34
Year = 2003	0.16 ± 0.32	0.11 ± 0.43	0.10 ± 0.43	−0.23 ± 0.30
Year = 2004	−0.56 ± 0.29	−0.73 ± 0.38	−0.73 ± 0.38	−0.70 ± 0.29
Year = 2005	0.03 ± 0.34	−0.22 ± 0.43	−0.22 ± 0.43	0.06 ± 0.35
Year = 2006	0.11 ± 0.31	−0.09 ± 0.43	−0.09 ± 0.43	0.05 ± 0.32
Year = 2007	−0.24 ± 0.31	−0.34 ± 0.41	−0.34 ± 0.41	−0.31 ± 0.31
Intercept	−1.27 ± 1.38	−0.32 ± 1.86	−0.29 ± 1.86	−1.36 ± 1.56
n-effective	6114	3473	3473	4237
*k*	15	13	14	14

The Date variable was centered prior to applying the quadratic transformation.

1 Standard errors for parameters for these models are inflated by the variance inflation factor *ĉ* (reported in notes for Table 1).

2 Parameters used to infer patterns referred to in column headings and reported in text or described in Figures 5 and 7.

### Experimental versus natural predation rates and patterns with respect to density and date

**Figure 6:**
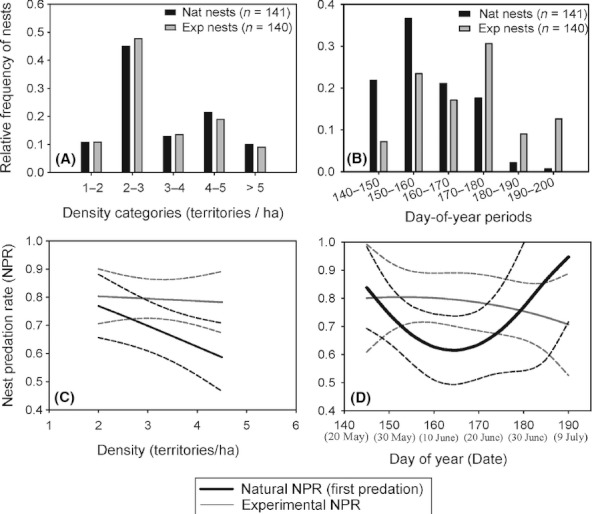
Comparison of experimental versus natural nest predation (NPR) patterns (2006–2007). Natural and experimental nests were monitored along a similar spatiotemporal extent (A, B). Continuous NPR estimates along Density axis (C) were calculated using M2 and along Date axis (D) using M7 (Set 1, Table 3) (parameter estimates in Table 4). Model estimates assume mean values for nontarget explanatory variables (Year = 0.125 for each level, PC1 = 0.1). Dotted lines show 95% confidence bands.

In 2006–2007, we monitored 111 experimental nests with >30% concealment and 139 natural nests during the egg period. Of these, 88 experimental nests (79.3%) were depredated, 68 natural nests (48.9%) were completely depredated, and 80 natural nests (57.6%) were either partially or completely depredated. Experimental and natural nests were monitored over similar territory density (experimental mean = 3.2 ± 1.2 [SD]; natural mean = 3.5 ± 1.2 territories/ha) and dates (experimental mean = 169 ± 14; natural mean = 160 ± 10 days from 1 January) (Fig. 6A and B). Throughout the entire study period (2001–2008), we monitored 590 natural nests during the egg period and we observed partial and/or complete predation of 336 clutches.

**Table 3:** Models fitted to data from natural and experimental nests monitored in 2006–2007 comparing natural versus experimental nest survival patterns

Model set no. (Dataset used)	Model no.	Models	*K*	−LL	Δ_*i*_	*w*_*i*_
Model set 1, Dataset NE_2_	1	Density	5	324.1	0.0	0.32
	2	Density + Type × Density	6	323.2	0.2	0.29
	3	Null model (Year + PC1 + Type)	4	325.5	0.9	0.21
	4	Constant survival	1	329.6	3.1	0.07
	5	No Type (Year + PC1)	3	327.7	3.2	0.06
	6	Date^2^	6	325.2	4.2	0.04
	7	Date^2^ + Type × Date^2^	8	324.2	6.2	0.01
Model set 2, Dataset NE_1_	1	Density + Type × Density	7	311.7	0.0	0.54
	2	Density	6	313.2	1.0	0.33
	3	Null model (Year + PC1 + Type + Parasitism)	5	315.8	4.2	0.07
	4	Date^2^ + Type × Date^2^	9	312.6	5.9	0.03
	5	Date^2^	7	315.2	6.9	0.02
	6	Constant survival	1	330.5	25.5	<0.01

*K* = number of model parameters, −LL = −Log-Likelihood, Δ_*i*_ = ΔAIC_c_, *w*_*i*_ = AIC_c_ weights. Only data from experimental nests >30% concealed were analyzed. Models were fitted to datasets for which natural nest failure was equated to first partial or complete predation (NE_2_, Fig. 4) or complete predation only (NE_1_, Fig. 4). Except for constant survival models, all models in each set contained a set of parameters that controlled for confounding effects (Year, PC1, and Nest Type for both model sets and Parasitism for model set 2). Parameter estimates for models used for inference are provided in Table 4.

Date^2^ = Date + Date^2^.

*ĉ* = 0.94 for Set 1 and *ĉ* = 0.95 for Set 2, calculated for a maximally parameterized model (Year + PC1 + Parasitism + Date^2^ + Date^2^ × Type + Density + Density × Type; not shown).

Overall, egg predation rates at natural nests were lower than experimental NPR even when controlling for differences between these nest types other than the presence of parents (i.e., Datasets NE_1–2_, Fig. 4). Regardless of how failure was defined for natural nests, the data supported a difference in natural- versus experimental nest survival rates (ER_M3/M5_ = 3.2, Set 1; ER_M3/M6_ = 4.3, Set 2; Table 3). When defining natural nest failure most comparably with experimental nest failure (i.e., first partial or complete predation), natural NPR_Egg_ (0.67 ± 0.04 [SE]) was substantially lower than experimental NPR (0.79 ± 0.04; derived from M3, Set 1, Table 3). Equating failure with complete predation, natural NPR_Egg_ was even lower (0.55 ± 0.05; calculated from DSR model with Year, PC1, Parasitism, and Nest-Type effects; for parameter estimates, see Table 4).

**Table 4:** Parameter estimates (β ± SE) for selected models (see Table 3) used for inferring differences in predation rates and patterns between natural and experimental Yellow Warbler nests (2006–2007)

Parameters	Density pattern (M3, Model set 1)	Date pattern (M7, Model set 1)	Nest type difference (M3, Model set 1)	Parasitism difference (model fitted to Dataset NE_2_, Figure 4)	Nest type and parasitism difference (M3, Model set 2)
Density	0.21 ± 0.10^1^	n/a	n/a	n/a	n/a
Date	n/a	0.000 ± 0.013^1^	n/a	n/a	n/a
Date^2^	n/a	−0.0015 ± 0.0011^1^	n/a	n/a	n/a
Nest Type = experimental	0.27 ± 0.47	0.82 ± 2.65	−0.35 ± 0.17^1^	−0.32 ± 0.19	−0.43 ± 0.20^1^
Type(Exp) × Density	−0.18 ± 0.14^1^	n/a	n/a	n/a	n/a
Type(Exp) × Date	n/a	0.001 ± 0.016^1^	n/a	n/a	n/a
Type(Exp) × Date^2^	n/a	0.0018 ± 0.0013^1^	n/a	n/a	n/a
Parasitism	n/a	n/a	n/a	0.09 ± 0.24^1^	0.72 ± 0.27^1^
PC1	−0.22 ± 0.08	−0.17 ± 0.08	−0.19 ± 0.08	−0.19 ± 0.08	−0.25 ± 0.09
Year = 2006	0.19 ± 0.17	0.10 ± 0.17	0.12 ± 0.16	0.12 ± 0.16	0.20 ± 0.17
Intercept	1.62 ± 0.36	2.51 ± 2.06	2.37 ± 0.15	2.45 ± 0.18	2.39 ± 0.18
n-effective	1513	1513	1513	1513	1671
*k*	6	8	5	5	5

The Date variable was centered prior to applying the quadratic transformation.

1 Parameters used to infer patterns referred to in column headings and reported in the text or presented in Figure 6.

Predation patterns recorded at natural nests differed from patterns recorded at experimental nests. Natural NPR decreased substantially with increasing territory density both in 2006 and 2007 (Fig. 6C) and throughout the entire study period (Fig. 5C), whereas experimental NPR did not vary with Density (Fig. 6C). Data from 2006 to 2007 provided only weak statistical support for a difference in Density effects (ER_M2/M1_ = 0.9, ER_M2/M3_ = 1.4, Set 1, Table 3). Nevertheless, when increasing our sample size by equating natural nest failure with complete predation, the data better supported the Density × Nest-Type interaction (ER_M1/M2_ = 1.6, ER_M1/M3_ = 8.1, Set 2; Table 3). Furthermore, when keeping the definition of natural nest failure comparable with experimental nest failure, naturalnest data throughout the study period (2001–2008) continued to support a negative relationship between territory density and predation rates (model set 2, Table 1; for relevant parameter estimates, see Table 4).

We found notable differences in Date-related patterns for natural versus experimental nests despite weak statistical support from 2006 to 2007 data. All natural-nest datasets described a similar mid-seasonal drop in NPR (Figs. 5B and D, 6D). Data from 2006 to 2007 (NE_1–2_, Fig. 4) failed to statistically support any seasonal effects on predation rates at all (ER_M6/M3_ = 0.2, ER_M7/M3_ = 0.1, Set 1; ER_M4/M3_ = 0.4, ER _M5/M3_ = 0.3, Set 2; Table 2). When natural nest failure was defined comparably with experimental nest failure, however, 2001–2008 egg-period data (N_3_, Fig. 4) provided stronger support for a Date + Date^2^ effect (Set 2, Table 1) and interannual variability in this pattern was not supported (ER_Date × Year model/Additive model_ = 0.01; the Additive model was M1, Set 2, Table 1). By contrast, experimental-nest data did not support a Date + Date^2^ effect (ER_M5/M1_ = 0.16, Set 1, Table 4), nor was there any suggestion of variation in experimental NPR over the nesting season (Fig. 6D). When controlling for the most differences between nest types (NE_2_, Fig. 4), mid-seasonal NPR at natural nests was lower than early- and late-season natural NPR, as well as experimental NPR (Fig. 6D; for relevant parameter estimates, see Table 4).

Observed patterns were not confounded with microhabitat effects on predation rates. By including the PC1 parameter, we controlled for confounding effects of microhabitat patch structure. Neither Density nor Date correlated strongly with Concealment for natural nests (Density: *r* = −0.21, Date: *r* = −0.13, *n* = 616 nests) nor for experimental nests (Density: *r* = −0.01, Date: *r* = 0.11, *n* = 111 nests), and scatter plots (not presented) did not suggest any nonlinear relationships. Furthermore, for experimental nests >30% concealed (i.e., the data included in this study), the concealment–predation relationship was weak (Latif et al. 2012), and unlike the Concealment × Year interaction effect found for natural nests (Latif et al. 2012), Density and Date effects did not interact with Year (both ER_Interaction-model/Additive-model_ < 0.01; Additive model = M1, Set 1, Table 1). Including the height parameter in DSR models did not unveil any seasonal patterns in experimental DSR (ER_M5/M1_ = 0.2, ER_M3/M1_ = 0.4, Set 1, Table 5), and scatter plots did not suggest any nonlinear relationships between height and Date. Furthermore, unlike the Height × PC1 relationship observed at natural nests (Latif et al. 2011), Density and Date effects did not interact with PC1 (both ERs_Interaction-models/Additive-model_ = 0.2; Additive model = M1, Set 1, Table 1). Despite a correlation between parasitism status and overall predation rates (described further below), parasitism was not confounded with Date- or Density-related predation patterns. Regardless of how nest failure was defined, ERs for spatiotemporal–Parasitism interactions were <1 (largest ER: ER_Date × Parasitism-model/Additive-model_ = 0.5; Additive model = Model 1, Set 1, Table 1), so differences in parasitism status could not have explained differences in natural versus experimental patterns.

**Table 5:** Daily nest-survival models fitted to data from experimental-nest data monitored 2006–2007 (E_1_, Fig. 4)

Model no.	Models	*K*	−LL	Δ_*i*_	*w*_*i*_
1	Null model (Year + PC1 + Height)	4	155.3	0.0	0.45
2	Age	5	155.2	1.7	0.19
3	Density	5	155.3	2.0	0.16
4	Age + Density	6	155.2	3.7	0.07
5	Date^2^	6	155.2	3.8	0.07
	^*^^*^^*^				
9	Constant survival	1	163.1	9.6	<0.01

*K* = number of model parameters, −LL = −Log-Likelihood, Δ_*i*_ = ΔAIC_c_, *w*_*i*_ = model weights. Only models with *w*_*i*_ > 0.05 and Constant Survival model shown. ^*^^*^^*^ indicates where additional models occurred but are not presented. Except for the constant survival model, all models contain parameters controlling for confounding effects (Year, PC1, and Height).

Date^2^ = Date + Date^2^.

*ĉ* = 0.31 for global model (M8; not shown).

### Age-related predation patterns

**Figure 7:**
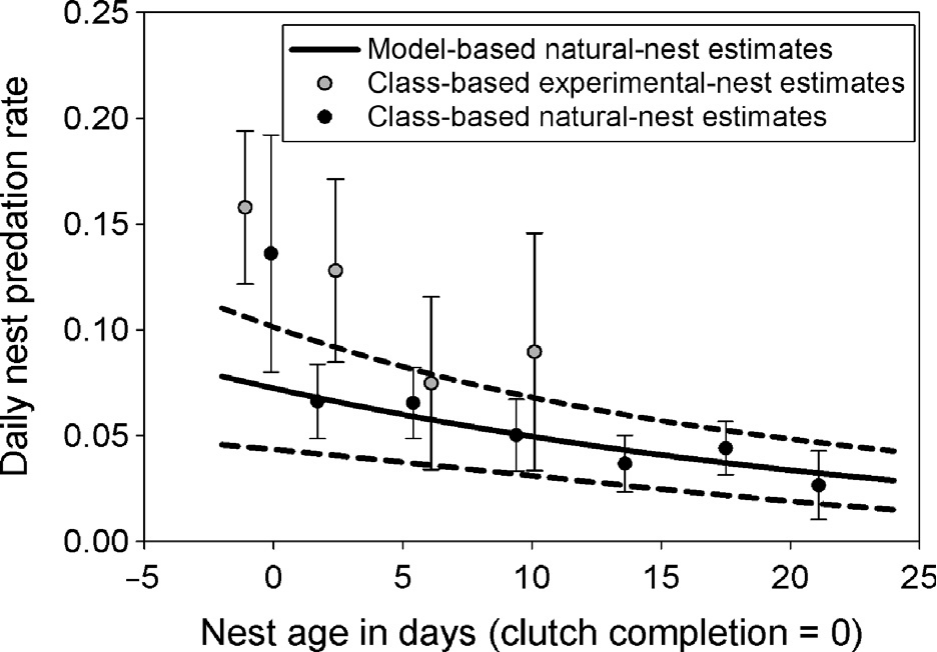
Daily nest predation rate estimates along an Age axis calculated using M1, Set 1, Table 6 (parameter estimates in Table 2). Dotted lines show 95% confidence bands. Model-based estimates assume mean values for nontarget explanatory variables (Year = 0.125 for each level, Date = 164, Date^2^ = 95, Parasitism = 0.4, PC1 = 0.2, Density = 3). Class-based estimates with 95% confidence intervals for natural (Black) and experimental nests (Gray) are plotted at mean Age values for observation intervals within each class. Class-based estimates for natural nests allow assessment of model fit (i.e., akin to plots of raw data alongside presentations of linear regression model predictions; Shaffer and Thompson 2007). Age effects were not statistically significant for experimental nests, but low statistical power was suspected within the egg period, so class-based estimates are presented to show possible trends. Variances for predation rates were calculated using the delta method (*var*(NPR) = *var*(NSR) = period^2^ × (DSR^2(period−1)^) × *var*(DSR); Powell 2007) and inflated by *ĉ* = 1.3.

Nest age was negatively correlated with natural NPR. Age was known in the field during 1418 natural-nest observation intervals (69% of all intervals). The continuous linear Age model best supported by these data (Table 6) described decreasing daily predation rates with nest age (Fig. 7). An age effect on experimental nest survival was not statistically supported (β_Age_ = 0.016 ± 0.030 in M2, Set 1, Table 4; ER_M2/M1_ = 0.42), but was also not supported within the natural-nest egg period (β_Age_ = 0.018 ± 0.027; ER = 0.45; from equivalent age models fitted to N_2_, Fig. 4). Class-based estimates, however, did suggest a decline in experimental daily nest predation rate with age comparable in magnitude to the apparent decline within the natural-nest egg period (Fig. 7).

**Table 6:** Models describing Age effects on daily nest survival fitted to data from 2001 to 2008 for which nest age (no. of days from clutch completion) was known in the field (N_2_, Fig. 4)

Model no.	Model	*K*	−LL	Δ_*i*_	*w*_*i*_
1	Age	14	594.1	0.0	0.44
2	Stage_Egg-or-Nestling_	14	594.8	1.3	0.23
3	Age + Age^2^	15	594.1	1.9	0.17
4	Stage_Laying-incubation-or-Nestling_	15	594.7	3.1	0.09
5	Age + Age^2^ + Age^3^	16	594.1	3.9	0.06
6	Null model	13	602.0	13.6	<0.01
7	Constant survival	1	625.9	39.5	<0.01

*K* = number of model parameters, −LL = −Log-Likelihood, Δ_*i*_ = ΔAIC_c_, *w*_*i*_ = model weights. Except for the constant survival model, all models included Date + Date^2^, Density, Parasitism, PC1, and Year to control for confounding effects. Parameter estimates for model 1 are provided in Table 2.

*ĉ* = 1.31 for M5, Set 1.

### Brood parasitism and nest predation

Natural NPR negatively correlated with cowbird parasitism. Natural-nest data supported a Parasitism relationship with complete-predation rates (ER_M1/M3_ = 5.2, Set 1, Table 1); parasitized nests suffered less predation (NPR = 0.71 ± 0.06 [SE]) than nonparasitized nests (NPR = 0.80 ± 0.05; estimated with M1, Set 1, Table 1; see parameter estimates in Table 2). Similarly, in 2006–2007, a parasitized natural nest was less likely to be completely depredated during the egg period (NPR = 0.41 ± 0.07) than a nonparasitized nest (0.65 ± 0.05; estimated with M3, Set 2, Table 3; parameter estimates in Table 4). This difference disappeared, however, when failure equaled either partial or complete predation (ER_(M3 + M4)/(M1 + M2)_ = 0.4, Set 2, Table 1). The likelihood of any predation was similar for parasitized (2001–2008: NPR_Egg_ = 0.91 ± 0.05; 2006–2007 NPR_Egg_: 0.66 ± 0.07) versus nonparasitized nests (2001–2008: NPR_Egg_ = 0.91 ± 0.06; 2006–2007: NPR_Egg_: 0.69 ± 0.05) (2001–2008 estimates from M3, Set 2, Table 1; 2006–2007 estimates from a Year + PC1 + Parasitism + Nest-Type model fitted to Dataset NE_1_, Fig. 4, and reported in Table 4). The difference in NPR when using the former versus the latter definition of failure provides an estimate of the probability of a nest being partially depredated prior to its final outcome (NPR_Partial_ = NPR_First-of-any_ − NPR_Complete_). From 2006 to 2007, parasitized clutches were more likely to be partially depredated (NPR_Partial_ = 0.25 ± 0.10) than nonparasitized clutches (NPR_Partial_ = 0.04 ± 0.09).

## Discussion

### Parents reduce the risk of nest predation

Our findings strongly suggest Yellow Warbler parents influenced NPR. When defining nest failure similarly for experimental and natural nests (i.e., first partial or complete predation), predation rate differences between nest types came closest to quantifying the parental effect. Our data suggest Yellow Warbler parents along Rush Creek reduced predation risk by approximately 12% during the 2006 and 2007 breeding seasons. Although experimental nests were never parasitized, natural NPR were not correlated with parasitism given a comparable definition of failure, so parasitism effects did not fully explain differences in natural versus experimental predation rates. Having controlled for microhabitat relationships, differences in predation rates between natural and experimental nests likely arose from postinitiation parental effects. In contrast, previous work recorded similar microhabitat-related patterns for natural and experimental nests, suggesting microhabitat relationships with predation risk were mainly driven by predator ecology (Latif et al. 2011) with some influence of nest-site selection on observed patterns (Latif et al. 2012). In so far as experimental NPR represent ambient levels of risk determined by nest-site quality and predator ecology, parents must have defended their nests in some way to reduce natural predation rates below this level. Yellow Warblers exhibit various defense behaviors, including active and passive defense (Lowther et al. 1999; Latif and Heath personal observations). A myriad of predators threaten songbird nests along Rush Creek (Latif et al. In press), and Yellow Warblers are probably capable of fending off at least some of these predators.

Cowbirds are important nest predators against which Yellow Warbler parents likely defend their nests. In addition to direct observations of cowbird predation at nests of other songbird species (Latif et al. In press), parasitism relationships with predation rates (i.e., higher complete-predation rates for nonparasitized nests, but higher partial-predation rates for parasitized nests) suggest predation of Yellow Warbler nests by cowbirds. Cowbirds may be less able to find nests that are depredated early, resulting in a negative parasitism–predation relationship. By coding parasitism status for each observation interval rather than for the entire nest period, however, we were able to control for any confounding effects of nest age. Selective parasitism of high-quality nest sites or hosts could also yield a negative correlation. We had no information on host quality, but parasitism did not correlate with known microhabitat correlates of nest survival (PC1: *r* = −0.03, Concealment: *r* = 0.01; *n* = 2060 observation intervals; for importance of these variables, see Latif et al. 2011, 2012). Trade-offs between host desirability and detectability could negate apparent microhabitat–parasitism relationships. An indirect measure of host detectability (hatching synchrony), however, was also unrelated with nest microhabitat in this system (Tonra et al. 2009). Evidence for direct predation by cowbirds does not negate the possibility that parasitism may also indirectly elevate predation risk for parasitized nests by conventional predators. Additional data and analyses are likely required to fully evaluate the impacts of cowbirds on Yellow Warbler fecundity (Zanette et al. 2007). Nevertheless, our data suggest the direct impacts of cowbirds removing host eggs and nestlings outweigh indirect impacts via increased nest activity at parasitized nests for Yellow Warblers in this system. Yellow Warblers exhibit specialized behaviors to prevent cowbirds from reaching their nests (Tewksbury et al. 2002; Gill and Sealy 2004), and anecdotal observations suggest small passerine birds can do so successfully (Strausberger 1998). Cowbirds were the most frequent predator identified with video cameras depredating experimental nests, but were never identified depredating Yellow Warbler nests (Latif et al. In press), suggesting potentially greater cowbird impacts if parents did not defend their nests.

### How variation in parental nest defense could drive predation patterns

Differences in natural versus experimental predation patterns indicate parents somehow contribute to these patterns. Spatiotemporal variability in parental behavior (required for pathway 2, Fig. 1) could arise from variation in food availability (Duncan Rastogi et al. 2006; Eggers et al. 2008). Food availability could modulate the amount of time parents invest in foraging, and consequently the remaining time left for nest defense (Martin 1992). For birds in North America, breeding densities generally correlate positively with fecundity (Bock and Jones 2004), probably because birds concentrate in high-quality habitats where food is abundant. Along Rush Creek, warblers were denser where willow was more prevalent (Density-PC1 correlation: *r* = 0.29, *n* = 169 territory values; derived from averaging 2006–2008 random-site scores for each territory). Given their higher foliage volume and occurrence in mesic sites (McBain and Trush 2003), willow shrubs likely provide valuable foraging opportunities for leaf-gleaning birds, such as Yellow Warblers. Indeed, along two other tributary streams of Mono Lake, Heath (unpubl. data) found that 21% of Yellow Warbler foraging attacks were in willow (second to 74% in black cottonwoods [*Populous balsamifera* spp*. trichocarpa*] which are rare in our Rush Creek study plots). In short, the variation in nest-survival rates that correlated with territory density may also correlate with food availability or some other habitat element related to food availability. Regardless, our results indicate some parental contribution to density-related variation in predation rates. Alternative to the food availability hypothesis, higher breeding densities could also allow cooperative nest defense (Hogstad 1995; Sperry et al. 2008). One might expect cooperative defense to yield area-wide predator deterrence and thus reduce predation rates for experimental nests (Andersson and Wiklund 1978). For noncolonial birds such as Yellow Warblers, however, parental alarm calls may be needed to enlist neighbors' assistance when predator attacks. Given the need for alarm calls to elicit cooperative defense, territory–density relationships with predation rates would only be apparent for natural nests. A seasonal peak in arthropod abundance could cause temporal variation in food availability capable of causing the apparent seasonal trough in predation rates. A peak in arthropod abundance was measured in 2010 at two other streams tributary to Mono Lake (Heath 2011), although the timing of this peak (mid-July) was not necessarily optimal for meeting the food requirements for parents that completed clutches on 20 June (i.e., the trough in predation rates). Measurement of arthropod abundance and, perhaps more importantly, foraging rates (Hutto 1990) concurrent with nest monitoring would be of interest in this system. Alternatively, seasonal variation in temperature could affect physiological energy balances of parents (Ardia et al. 2009), which could in turn affect relative investments in foraging versus nest defense.

Variation in parental interactions among predator species that differ in their relationships with environmental or temporal factors could also influence patterns of predation risk (pathway 3, Fig. 1). The predator species responsible for causing density- and date-related patterns observed at natural nests should be those that are relatively resistant to parental defense, and therefore depredate natural nests more frequently than experimental nests. Cowbirds are likely important predators of natural nests, but they were also likely frequent predators of experimental nests (Latif et al. In press). Nevertheless, considering the complexity of behavioral interactions between cowbirds and their hosts (e.g., in addition to other studies cited above and below, see Robinson and Robinson 2001; Guigueno and Sealy 2012) the role of cowbirds in producing observed patterns may be worth further examination. Although frequent predators of nestlings, snakes were never observed depredating eggs in this system (Latif et al. In press), Nevertheless, egg predation by snakes has been documented (Thompson and Burhans 2003) where snakes did not depredate artificial nests (Thompson and Burhans 2004). Individual snakes can grow fairly large and may therefore be difficult for parents to fend off once the nest has been discovered. Additionally, snake ecology did correlate with temporal nest predation patterns in a Midwestern bird community (Weatherhead et al. 2010). At least two types of rodents, chipmunks and mice, depredated songbird nests along Rush Creek and also depredated experimental nests less frequently than avian predators (see clay-egg bite data reported by Latif et al. 2011, 2012, In press). If rodents, snakes or cowbirds drive observed spatiotemporal predation patterns, results from this study indicate that parental activity is required for these patterns to emerge. Therefore, factors affecting parental behavior (e.g., food availability or temperature) would likely modulate the strength of these patterns if not directly drive them.

### Why predation risk decreases with nest age

In contrast with Density- and Date-related patterns, age-related variation in predation rates did not appear to involve parents, suggesting predator ecology is mainly responsible for this pattern (pathway 1, Fig. 1). Similarities in microhabitat-related patterns for natural versus experimental nests (Latif et al. 2011, 2012) also suggest predator ecology as the primary driver. Variation in predation risk among nest sites can cause a positive age relationship (as observed here) when nests in poor-quality nest sites are depredated quickly leaving only nests in low-risk sites to reach older ages (Dinsmore et al. 2002). Variation in parental behavior can also cause positive age relationships with predation risk (Andersson and Waldeck 2006), but parental effects could not influence experimental predation patterns, which appeared consistent with natural age-related patterns in this study. Our results are consistent with those of Martin et al. (2000), who demonstrated the need to control for microhabitat effects to document effects of increased nest activity later in the nesting cycle.

### Limitations and advantages of experimental nests

The strength of our inferences depends both on how well we controlled for differences between natural and experimental nests and on whether differences for which we could not control provide alternative explanations of observed patterns. The two nest types were monitored using the same field protocols, so we controlled for observer influence on cues leading predators to nests (e.g., scent trails, time at nests, number of nest visits). We had less control over differences in sensory cues at the nest site. Unattended, nonviable eggs may rot faster, providing additional olfactory cues that could attract predators. Storage protocols (DeGraaf and Maier 2001) minimized rotting of eggs prior to their deployment, and eggs that avoided predation did not show any obvious signs of rot when retrieved from the field. Sensory cues provided by parents could also attract predators (Ghalambor and Martin 2002), but if this were the case in our study, natural NPR should have been higher than experimental predation rates. Parents could both attract predators and defend against them, in which case our data would indicate an even stronger parental-defense effect than was apparent from our analysis (i.e., the difference between natural and experimental nests plus the attractant effect). In addition to the reasons described above, cowbirds may depredate eggs to assess their incubation status and thus inform parasitic decisions (Massoni and Reboreda 1999). Cowbirds may also use parental behavior to assess a nest's status and therefore become less apt to depredate eggs in active nests that provide this cue. Given this scenario, spatiotemporal variation in cues provided by parents to cowbirds could explain predation patterns observed at natural nests. In short, differences for which we did not control are either unlikely to play a prominent role in shaping observed patterns or unlikely to negate our principal conclusion that parents are a necessary component of mechanisms underlying observed patterns. Additional data describing parental behavior at nests would be beneficial for corroborating our conclusions. Nevertheless, experimental manipulation of parental behavior may be more difficult and is not ethical at the level afforded by experimental nests (i.e., complete removal of parents). Thus, despite their limitations, experimental nests may provide information about parental effects on nest predation not afforded by other methods.

### Further implications

Although Yellow Warblers are a relatively common species in North America, they are a species of conservation concern in California having been largely extirpated from the Central Valley and other localized areas (Heath 2008). Furthermore, the population health of this species is considered an indicator of the more general health of riparian systems (RHJV 2004). In addition to components of the environment that influence nest predator ecology, results from this study indicate the potential importance of factors affecting parental behavior for preserving fecundity levels requisite for continued population persistence.

Previous work documented Yellow Warbler preferences for high-predation willow-dominated nest microhabitats (Latif et al. 2011), raising two questions: (1) why do Yellow Warblers favor higher predation microhabitats, and (2) how do these choices influence population persistence? If non-microhabitat choices positively influence fecundity, selection of low-predation nest microhabitats may be less important for achieving high fitness and positive population growth. In contrast with microhabitat choices, Yellow Warblers in this study concentrated their territories where predation rates were low. Furthermore, the difference in predation rates between the least-populated and most-populated areas was similar in magnitude to the difference between preferred versus less-preferred nest microhabitats (compare Fig. 5A here with Fig. 1B in Latif et al. 2011). Willow was positively correlated with territory density, so in contrast with its influence at the microhabitat scale, willow at the territory scale may be unrelated or negatively correlated with predation rates. The optimal habitat-selection strategy for maximizing nest survival may be to favor willow-dominated territories, but avoid willow when selecting nest sites. Nevertheless, selection of high-quality territories alone may be sufficient to attain high enough nest-survival rates for positive fitness and population persistence. Nesting early and often should also benefit fecundity, especially since nest-survival rates improve following early nest failure, at least initially. Population models could help elucidate the relative influence of different nest-survival correlates on fecundity, and thus which decisions made by Yellow Warblers are most important for maximizing fitness and population growth.

Although the potential for parents to influence nest survival has been recognized (Martin 1992), the importance of predator ecology is more widely recognized (Thompson 2007). This study demonstrates the potential importance of parents for influencing predation patterns and a readily available approach for examining the contribution of parents.
